# Effects of regional citrate anticoagulation on thrombin generation, fibrinolysis and platelet function in critically ill patients receiving continuous renal replacement therapy for acute kidney injury: a prospective study

**DOI:** 10.1186/s13613-022-01004-w

**Published:** 2022-03-31

**Authors:** Richard Fisher, Gary W. Moore, Michael J. Mitchell, Letian Dai, Siobhan Crichton, Nuttha Lumlertgul, Marlies Ostermann

**Affiliations:** 1 Department of Critical Care, King’s College Hospital, London, UK; 2 Department of Haemostasis and Thrombosis, Viapath Analytics LLP, Guy’s & St Thomas’ NHS Foundation Trust, London, UK; 3 Faculty of Science and Technology, Middlesex University, London, UK; 4 MRC Clinical Trials Unit, University College London, London, UK; 5 Department of Critical Care, King’s College London, Guy’s & St Thomas’ Hospital, London, SE1 7EH UK; 6 Division of Nephrology, Department of Internal Medicine, King Chulalongkorn Memorial Hospital, Bangkok, Thailand; 7 Excellence Centre in Critical Care Nephrology, King Chulalongkorn Memorial Hospital, Bangkok, Thailand; 8 Critical Care Nephrology Research Unit, Chulalongkorn University, Bangkok, Thailand

**Keywords:** Acute kidney injury, Citrate, Clotting, CRRT, Continuous renal replacement therapy, Thrombin, Platelets

## Abstract

**Background:**

Regional citrate anticoagulation (RCA) is recommended for continuous renal replacement therapy (CRRT). However, filter life varies and premature filter clotting can occur. The aims of this explorative prospective study were to investigate the effects of RCA on thrombin generation, fibrinolysis and platelet function in critically ill patients receiving CRRT, to compare clotting parameters between systemic and intra-circuit blood samples, and to screen participants for coagulation disorders. We recruited critically ill adult patients admitted to a 30-bedded Intensive care unit in a tertiary care hospital who required CRRT with RCA for acute kidney injury (AKI). Patients with pre-existing thrombotic, bleeding tendencies or a CRRT duration less than 48 h were excluded. We measured coagulation and thrombophilia parameters at baseline. Thrombin generation, D-dimer and platelet function were measured pre-CRRT and at 12, 24, 36, 48 and 72 h after commencing CRRT using blood samples taken from the arterial line and the circuit.

**Results:**

At baseline, all eleven patients (mean age 62.4 years, 82% male) had Factor VIII and von Willebrand Factor concentrations above reference range and significantly increased peak thrombin generation. During CRRT, there were no significant changes in systemic maximum peak thrombin generation, time to peak thrombin generation, fibrinogen, D-dimer and platelet function analysis. We observed no significant difference between paired samples taken from the patient's arterial line and the circuit.

**Conclusions:**

Critically ill patients with AKI requiring CRRT are hypercoagulable. Citrate used for anticoagulation during CRRT does not affect thrombin generation, D-dimer or platelet function. Systemic clotting parameters reflect intra-circuit results.

*Trial registration*: ClinicalTrials.gov Identifier: NCT02486614.

Registered 01 July 2015—Registered after recruitment of first patient. https://clinicaltrials.gov/ct2/show/NCT02486614

**Supplementary Information:**

The online version contains supplementary material available at 10.1186/s13613-022-01004-w.

## Introduction

Acute kidney injury (AKI) is a common complication during critical illness and renal replacement therapy (RRT) is often required [1–3]. Regional citrate anticoagulation (RCA) is recommended to maintain filter patency during continuous renal replacement therapy (CRRT) [4–7]. Citrate acts by chelating calcium and inhibiting calcium-dependent steps of clotting biochemistry [8]. Although filter life is significantly better with RCA compared with systemic heparin [7, 9–11], premature filter clotting can occur despite post-filter ionised [Ca^2+^] in the target range [12, 13]. Potential explanations for reduced filter life are sub-optimal vascular access and the existence of conditions that are associated with hypercoagulability. It has also been suggested that systemic haemostasis results do not always reflect coagulation parameters within the circuit and may lead to inappropriate clinical decision-making [14]. Whether citrate also impacts thrombin generation, fibrinolysis or platelet function and impacts filter life through these mechanisms is unknown [15–17]. Until recently, there were no data to this effect. In June 2021, Wiegele et al. reported the results of an observational study in 24 critically ill patients receiving citrate-based continuous veno-venous haemodialysis (CVVHD) [18]. They showed no significant changes in thrombin generation and platelet function after start or during CVVHD. The impact on fibrinolysis was not investigated. Also, a proportion of patients received blood transfusions, platelet transfusions, fresh frozen plasma and/or fibrinogen concentrates during the study period. Patients were not screened for any underlying coagulopathies.

The objectives of our explorative prospective study were.

(i) to investigate thrombin generation, fibrinolysis and platelet function in critically ill patients receiving CRRT with RCA who were not known to have a coagulopathy or any confounding comorbidities;
(ii) to compare haemostasis results between systemic and intra-circuit blood samples, and
(iii) to screen all participants for any potential unrecognised coagulopathies.

## Results

### Patient cohort

**Fig. 1:**
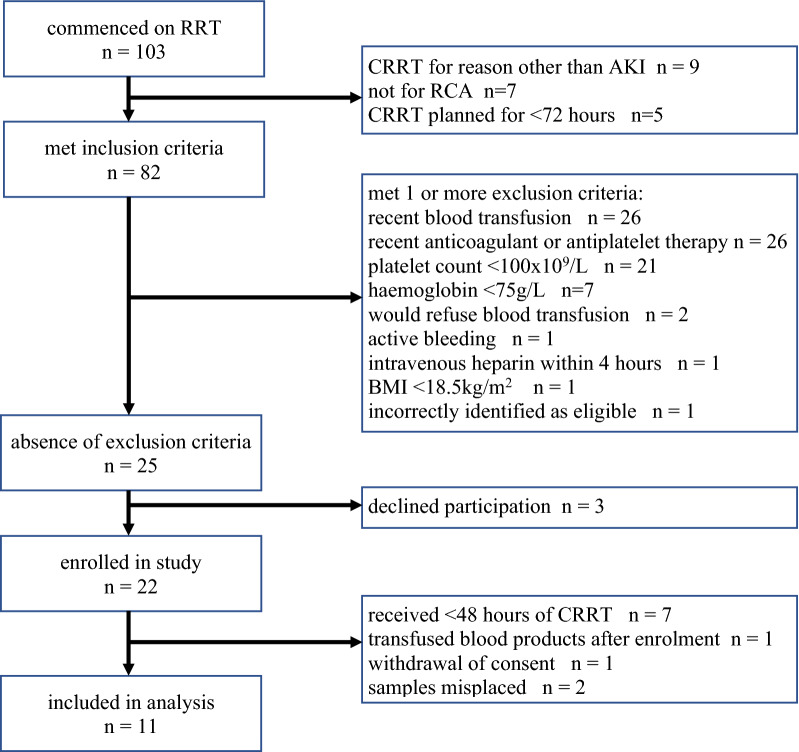
Cohort selection flowchart. *BMI* body mass index, *CRRT* continuous renal replacement therapy, *RCA* regional citrate anticoagulation

A total of 103 patients were screened of whom 22 were enrolled. Following exclusion of 11 patients (Fig. 1), 11 participants were included in the final analysis (mean age 62.4 ± 15.2 years; 81.8% male) (Table 1). Nine patients were treated with CRRT for > 72 h of whom 4 received un-interrupted CRRT and 5 had interruptions in therapy (receiving a mean of 67.8 h of CRRT throughout the 72-h study period). Two patients had CRRT permanently discontinued before 72 h had elapsed (both received CRRT for 65 h).

**Fig. 2:**
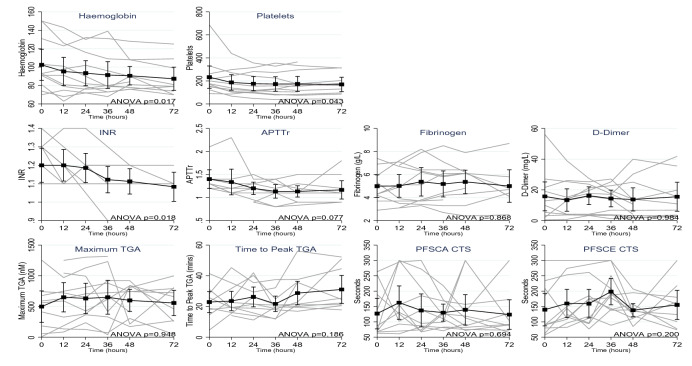
Changes in systemic measurements of coagulation over duration of treatment. *INR* International Normalised Ratio, *APTTr* activated partial thromboplastin time ratio, *TGA* thrombin generation assay, *PFSCA* platelet function assay using collagen/adenosine diphosphate, *PFSCE* platelet function assay using collagen/epinephrine, *CT* closure time. Repeated measures ANOVAs compared systemic parameters from baseline to 48 h. Post hoc pairwise comparisons found significant differences between haemoglobin at baseline and 36 h (*p* = 0.030) and baseline and 48 h (*p* = 0.018); platelets at baseline and 36 h (*p* = 0.035) and baseline and 48 h (*p* = 0.040) and INR at baseline and 36 h (*p* = 0.034) and baseline and 48 h (*p* = 0.024). There were no significant differences in systemic parameters on CRRT

**Fig. 3:**
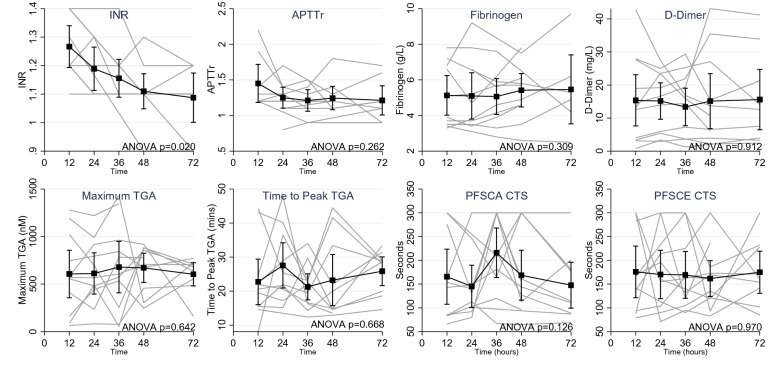
Changes in circuit measurements of coagulation over duration of treatment. *INR* International Normalised Ratio, *APTTr* activated partial thromboplastin time ratio, *TGA* thrombin generation assay, *PFSCA* platelet function assay using collagen/adenosine diphosphate, *PFSCE* platelet function assay using collagen/epinephrine, *CT* closure time. Repeated measures ANOVAs of measurements from the circuit compared data from 12 to 48 h. Only INR differed overtime, with a significant difference between 12 and 48 h in post hoc comparisons (*p* = 0.002)

**Fig. 4:**
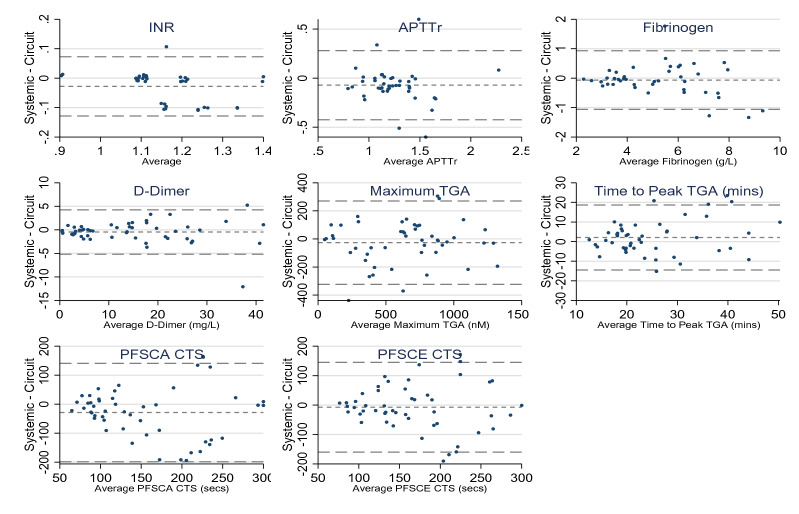
Bland–Altman plots showing difference between systemic and circuit measurements. *INR* International Normalised Ratio, *APTTr* activated partial thromboplastin time ratio, *TGA* thrombin generation assay, *PFSCA* platelet function assay using collagen/adenosine diphosphate, *PFSCE* platelet function assay using collagen/epinephrine, *CT* closure time. Lines shown represent the mean differences in systemic and circuit measurements and 95% limits of agreement

**Table 1:** Baseline characteristics of patients included in analysis

Baseline characteristics	Values
Age, years (mean, SD)	62.4 (15.2)
Male sex (n, %)	9 (81.8%)
BMI, kg/m^2^ (mean, SD)	30.9 (8.5)
Reason for critical care admission (n)	
Sepsis	3
Severe pancreatitis	4
Post-surgery	2
Trauma	1
Stroke	1
Comorbidities (n)	
Diabetes	3
Hypertension	4
APACHE II score (mean, SD)	20.1 (3.0)
SOFA score (mean, SD)	10.1 (2.7)
Mechanical ventilation (n, %)	9 (81.8%)
Vasopressor (n, %)	8 (72.7%)
Laboratory parameters pre-CRRT (mean, SD)	
White cell count, × 10^9^/L	21.0 (10.4)
Neutrophils, × 10^9^/L	18.1 (10.2)
Urea, mmol/L	17.0 (6.0)
Creatinine, µmol/L	280.2 (105.0)
Albumin, g/L	24.5 (4.0)
C-reactive protein, mg/L	208.2 (113.6)

*APACHE II* Acute Physiologic and Chronic Health Evaluation II, *BMI* body mass index, *CRRT* continuous renal replacement therapy, *SD* standard deviation, *SOFA* Sequential Organ Failure Assessment

During CRRT, 164 measurements of post-filter ionised [Ca^2+^] were undertaken by the clinical staff as per departmental protocol to ensure adequacy of anticoagulation. The ionised [Ca^2+^] was within target range on all but 4 occasions where [Ca^2+^] was > 0.34 mmol/L and citrate administration was adjusted as per protocol.

### Haemostasis assessment

#### Baseline assessment

Analysis of baseline samples (pre-CRRT) confirmed evidence of hypercoagulability which was not known to the clinical or research team prior to enrolment. (Table 2) Factor VIII concentrations and von Willebrand factor (VWF) were markedly above the reference range in all patients. In addition, 7 of 8 patients had elevated thrombin:antithrombin complexes (TAT), and in 9 of 11 patients protein C activity was below the reference range. Lupus anticoagulant was detected in 5 of 11 patients, 3 of whom also had elevated anticardiolipin antibodies and/or antiβ2-glycoprotein I antibodies. Functional activated protein C resistance (APCR) assays suggested that 2 patients had acquired APCR, both of whom had elevated Factor VIII concentrations (Fig. 2).

**Table 2:** Summary of measurements at baseline (pre-CRRT)

Investigations	Units	Reference range	Number with abnormal results	Range of abnormal results	Mean/median
Thrombin time	Ratio	(0.86–1.14)	1	1.19	N/A
Classic APCR	Ratio	(3.3–5.82)	3	2.22, 2.25, 2.44	2.30/2.25
Modified APCR	Ratio	(2.63–3.34)	3	1.81,2.51, 2.53	2.28/2.51
Antithrombin activity	IU/dL	(85.9–117.5)	9	24.2–80.0	61.8/67.4
Protein C activity	IU/dL	(77.1–126.4)	9	38.5–72.5	58.3/61.5
Free protein S antigen	IU/dL	(80.2–137.4)	5	48.9–76.2	67.7/70.4
Lupus anticoagulant by dRVVT and dAPTT	-	Not detected	5	5 × positive	N/A
IgG aCL	GPL U/mL	(0–10)	3	11.2, 13.5, 17.2	14.0/13.5
IgM aCL	MPL U/mL	(0–10)	3	19.2, 19.7, 31.9	23.6/19.7
IgG aβ2GPI	SGU	(0–2.9)	3	3.9, 6.0, 8.6	6.2/6.0
IgM aβ2GPI	SMU	(0–6.5)	7	6.8–31.9	15.0/12.0
Homocysteine	µmol/L	(0–15)	5	15.2–18.0	16.1/15.6
VWF:GPIbR	IU/dL	(41–127)	4	380.1–494.1	426.0/415.0
VWF:Ag	IU/dL	(50–172)	11	280.8–682.7	531.3/581.3
FVIII:C	IU/dL	(50–150)	11	199.4–614.4	418.4/432.6
Thrombin:antithrombin complexes (TAT)	µg/L	(1–4.1)	7	9.22–74.6	24.3/25.1
Prothrombin fragment 1.2	pmol/L	(69–229)	3	333.3, 401.1, 964.0	566.1/401.1
Prothrombin 20,210	–	–	1	1 × heterozygous	N/A
Factor V Leiden	–	–	0	–	–

*aβ2GPI* anti-β2 glycoprotein I antibodies, *aCL* anticardiolipin antibodies, *APCR* activated protein C resistance, *dAPTT* dilute activated partial thromboplastin time, *dRVVT* dilute Russell’s viper venom time, *FVIII:C* factor VIII coagulant activity assay, *VWF:GPIbR* ristocetin-triggered von Willebrand factor glycoprotein Ib-binding activity assay, *VWF:Ag* von Willebrand factor antigen

In TGA analysis, the mean peak thrombin was 498.5 nM (SD 390.6) (range 7.8–1255.7 nM), which was significantly greater than the mean of the control group (*p* = 0.002). Mean time to peak was 22.9 min (SD 10.5) (range 4.7–41.4 min) which was statistically not significantly different from time to peak in the control group (*p* = 0.26). Mean PFA-100 CT was 127 s (SD 80) (reference range 63-263 s) using the collagen/ADP cartridge (PFSCA) and 122 s (SD 68) (reference range 57–234 s) using the collagen/epinephrine cartridge (PFSCE). Mean INR and APTTr values were also elevated at baseline.

#### Measurements during CRRT

We observed a significant decline in haemoglobin (*p* = 0.017), platelet count (*p* = 0.043) and systemic INR (*p* = 0.018) during the 72-h period of CRRT, while systemic APTTr showed a statistically non-significant trend of decline (*p* = 0.077). There was no significant variation in systemic maximum peak thrombin in TGA, time to peak in TGA, fibrinogen, D-dimer and PFA-100 CT (PFSCA and PFSCE) (Table 3, Additional file 1: Figure S1).

**Table 3:** Changes in measurements of coagulation over duration of treatment

	Baseline	12 h	24 h	36 h	48 h	72 h	*p *value*
Haemoglobin (g/L)	102 (28)	95 (26)	93 (23)	91 (25)	91 (17)	87 (19)	0.017
Haematocrit	0.32 (0.09)	0.29 (0.08)	0.29 (0.07)	0.28 (0.08)	0.28 (0.06)	0.27 (0.06)	
Platelets (× 10^9^/L)	233 (164)	187 (114)	176 (104)	173 (107)	174 (110)	169 (95)	0.043
INR							
Systemic	1.18 (0.15)	1.13 (0.13)	1.13 (0.13)	1.10 (0.11)	1.08 (0.10)	1.06 (0.09)	0.018
Circuit		1.22 (0.15)	1.15 (0.13)	1.13 (0.11)	1.10 (0.10)	1.08 (0.12)	0.020
APTTr							
Systemic	1.29 (0.31)	1.25 (0.37)	1.18 (0.20)	1.11 (0.21)	1.11 (0.18)	1.17 (0.30)	0.077
Circuit		1.33 (0.38)	1.23 (0.24)	1.17 (0.22)	1.20 (0.24)	1.19 (0.28)	0.262
Fibrinogen (g/L)							
Systemic	4.81 (1.47)	5.01 (1.67)	5.53 (1.85)	5.25 (1.74)	5.36 (1.64)	5.13 (1.80)	0.868
Circuit		5.12 (1.70)	5.27 (2.07)	5.33 (1.86)	5.42 (1.58)	5.31 (1.93)	0.309
D-Dimer (mg/L)							
Systemic	15.8 (15.9)	13.4 (11.8)	16.2 (9.3)	14.4 (9.2)	13.8 (12.8)	15.5 (14.5)	0.984
Circuit		15.4 (13.1)	15.2 (9.3)	13.4 (9.2)	15.1 (14.1)	15.6 (13.9)	0.912
TGA: maximum thrombin generation (nM)**							
Systemic	498.5 (390.6)	650.2 (383.8)	631.5 (397.7)	649.9 (443.9)	599.3 (290.3)	557.3 (315.1)	0.948
Circuit		606.4 (422.9)	612.2 (349.7)	678.9 (458.3)	670.8 (249.0)	603.5 (187.6)	0.642
TGA: time to peak thrombin generation (min)**							
Systemic	22.9 (10.5)	21.5 (11.2)	26.3 (10.4)	21.7 (8.1)	28.7 (12.4)	31.1 (14.0)	0.186
Circuit		22.7 (11.3)	27.6 (10.7)	21.3 (6.5)	23.2 (12.1)	25.9 (6.5)	0.668
PFSCA CT (s)***							
Systemic	127(80)	162 (92)	137 (82)	130 (45)	139 (84)	123 (74)	0.694
Circuit		166 (94)	145 (72)	216 (84)	169 (89)	148 (74)	0.126
PFSCE CT (s)****							
Systemic	140 (85)	160 (77)	159 (75)	198 (69)	138 (36)	155 (73)	0.200
Circuit		176 (87)	170 (82)	169 (80)	162 (64)	175 (68)	0.970

*INR* International Normalised Ratio, *APTTr* activated partial thromboplastin time ratio, *TGA* thrombin generation assay, *PFSCA* platelet function assay using collagen/adenosine diphosphate, *PFSCE* platelet function assay using collagen/epinephrine, *CT* closure time

^*^Repeated measures ANOVAs compared systemic parameters from baseline to 48 h. Post hoc pairwise comparisons found significant differences between haemoglobin at baseline and 36 h (*p* = 0.030) and baseline and 48 h (*p* = 0.018); platelets at baseline and 36 h (*p* = 0.035) and baseline and 48 h (*p* = 0.040) and INR at baseline and 36 h (*p* = 0.034) and baseline and 48 h (*p* = 0.024). There were no significant differences in systemic parameters on CRRT. Repeated measures ANOVAs of measurements from the circuit compared data from 12 to 48 h. Only INR differed overtime, with a significant difference between 12 and 48 h in post hoc comparisons (*p* = 0.002)

^**^Test failed to return a result in 6.8% of samples (8/117)

^***^Test exceeded maximum permitted duration in 25.6% of samples (30/117); lower bound for duration of 300 s was used in analysis

^****^Test exceeded maximum permitted duration in 27.4% of samples (32/117), lower bound for duration of 300 s was used in analysis

INR results of samples taken from the circuit were significantly lower at 72 h compared to first sample taken during CRRT (*p* = 0.020) (Table 3, Additional file 1: Figure S2). No significant changes of the circuit APTTr, fibrinogen, D-dimer, maximum TGA, time to peak TGA, and PFA-100 results were observed over time (Fig. 3).

### Comparison between systemic and intra-circuit samples

Table 4 shows mean differences and limits of agreement between systemic and intra-circuit samples. INR, APTTr and PFSCA results were significantly higher in blood samples drawn directly from the circuit than in systemic samples taken from the patient's arterial line but there was no significant difference between paired fibrinogen, D-dimer, maximum TGA, time to peak TGA, and PFSCE results (Additional file 1: Figure S3).

**Table 4:** Agreement between systemic and circuit measurements

	Mean difference *	95% CI for mean difference		SD difference	Limits of agreement	
		Lower limit	Upper limit		Lower limit	Upper limit
INR	− 0.03	− 0.05	− 0.01	0.05	− 0.13	0.07
APTTr	− 0.07	− 0.13	− 0.02	0.18	0.42	0.28
Fibrinogen (g/L)	− 0.06	− 0.22	0.09	0.51	− 1.06	0.93
D-Dimer (mg/L)	− 4.5	− 0.15	0.21	2.4	− 5.2	4.2
TGA: maximum thrombin generation (nM)	− 26.6	− 70.2	16.9	151.6	− 323.7	270.4
TGA: time to peak thrombin generation (mins)	2.1	− 0.4	4.6	8.5	− 14.5	18.7
PFSCA CT (s)	− 28.8ρ	− 53.4	− 4.2	86.6	− 198.5	140.9
PFSCE CT (s)	− 7.4	− 29.5	14.7	77.7	− 159.7	145.0

*APTTr* activated partial thromboplastin time ratio, *INR* International Normalised Ratio, *TGA* thrombin generation assay, *PFSCA* platelet function assay using collagen/adenosine diphosphate, *PFSCE* platelet function assay using collagen/epinephrine, *CT* closure time, *CI* confidence interval, *SD* standard deviation

^*^Average difference between results of systemic samples and intra-circuit samples

## Discussion

The main findings of this study are: (i) citrate-based anticoagulation does not impact thrombin generation, platelet function or fibrinolysis during CRRT; (ii) critically ill patients with severe AKI are hypercoagulable with laboratory evidence of coagulopathies that were not known to the clinical team, and (iii) there was no significant difference in the coagulation results between systemic blood samples and samples drawn directly from the circuit (Fig. 4).

These results are important for clinical practice for the following reasons: filter life during citrate-based CRRT can be variable even when post-filter [Ca^2+^] is in target range. It is reassuring to know that this is not due to effects of citrate on thrombin generation or platelet function or inhibition of fibrinolysis. The fact that there was no significant difference between systemic and circuit samples provides re-assurance, too. Our data confirm that critical illness associated hypercoagulability is very common.

Despite the fact that we excluded all patients with a known bleeding or clotting diathesis and also patients with premature filter clotting, we found markedly raised factor VIII concentrations and VWF in all patients. TAT and prothrombin fragment 1.2 were increased in the majority of patients. Most patients also had reduced levels of protein C which most likely reflects the severity of critical illness rather than hereditary deficiencies. Some patients had positive lupus anticoagulant and mildly raised anticardiolipin and anti-β2 glycoprotein I antibody levels. Without checking for persistence of the antibodies, it is not possible to suggest the possibility of pre-event antiphospholipid syndrome. Two of five patients had low classic APCR ratio and only slightly reduced Modified APCR ratio, which reflects acquired APCR, most likely due to the grossly elevated FVIII levels and/or the lupus anticoagulants. One patient had reduced Classic APCR ratio accompanied by markedly reduced Modified APCR ratio, indicating hereditary APCR, although genetic analysis for FV Leiden, the most common *F5* variant conferring APCR, was not performed [19].

Thrombin, a key protein in the regulation of haemostatic processes, has both pro- and anticoagulant properties. TGAs evaluate thrombin generation and decay. The peak represents the highest thrombin concentration that can be generated, and the time to reach the peak represents the velocity of thrombin generation. In contrast, PT and APTT indicate whether there is a coagulation deficiency of one or more procoagulant factors, but not whether this deficiency is counterbalanced by a concomitant deficiency of anticoagulant factors. Our results showed that pre-CRRT, thrombin generation was significantly higher and faster in patients than in healthy volunteers, supporting the concept of critical illness associated hypercoagulability. Data on TGA in critically ill patients have previously been reported in the context of liver failure, trauma, sepsis, severe burns and extracorporeal membrane oxygenation, but to the best of our knowledge, the report by Wiegele et al. and our data are the first in patients with severe AKI requiring RRT [18, 20–22].

During CRRT with RCA, there was a normalisation of INR and APTTr from baseline but no significant change in D-dimer, fibrinogen, and peak and time to peak thrombin generation. It is unlikely that the normalisation of INR and APTTr reflects increased activation of coagulation as it would have been accompanied by a decrease in time to peak, an increase in peak thrombin, and possibly further D-dimer elevation.

To test platelet function, we used the PFA-100 analyser which is based on the property of platelets to adhere to collagen via VWF under conditions of shear stress and to aggregate in the presence of agonists [23]. The method involves the drawing of citrate anticoagulated blood through a narrow bore capillary that has at its end a collagen-coated membrane in which a defined microscopic aperture (147 μm) is present. Platelets adhere to the collagen which is infused with either adenosine diphosphate (ADP) or epinephrine to stimulate aggregation. A platelet clot occurs because of shear stress and agonists. The time taken by platelets to occlude the orifice and to block the flow is defined as the closure time (CT). During the 72-h period on CRRT, we observed no significant change in CT using ADP or collagen/epinephrine. We also did not detect any significant changes in systemic or intra-circuit D-dimer concentrations which suggests that fibrinolysis was not affected by citrate either.

This study adds to the existing data on the mechanisms of citrate and complements the findings by Wiegele et al. who studied 24 critically ill surgical patients and showed that thrombin generation did not change during citrate-based CVVHD [18]. Using multiple electrode aggregometry, they also demonstrated decreased platelet function at baseline and during CVVHD but citrate had no impact. A different study focused on the mechanisms of early filter clotting and compared the effects of citrate, heparin, and no anticoagulation strategies on TAT, activated protein C-protein C inhibitor (APC-PCI), and PAI-1 [14]. It showed that in case of early filter failure (< 24 h), inlet concentrations of TAT and APC-PCI were higher, irrespective of anticoagulation. In the heparin group, there was more production of APC-PCI and platelet-derived PAI-1 in the filter after 10 min than in patients who received citrate. Another study explored coagulation parameters in critically ill patients with AKI receiving CRRT with heparin or RCA [24]. Patients with active bleeding had RCA, whereas those without bleeding received heparin anticoagulation. Pre-existing coagulopathy or bleeding disorder was not an exclusion criterion. The study demonstrated no changes in platelets, TAT complexes, beta-thromboglobulin, and VWF during RCA.

An important strength of our analysis is the application of strict eligibility criteria to minimise the impact of confounding factors and the exclusion of participants who needed blood products, had CRRT for less than 48 h or developed premature filter clotting whilst in the study. This allowed us to investigate the direct impact of citrate as best as possible. In addition, citrate-based CVVHD was delivered by an experienced clinical team according to an established protocol [13]. Finally, the analyses were undertaken in a Centre for Haemostasis and Thrombosis at a tertiary care centre using established techniques. The data will serve to underpin future research studies and quality improvement projects in critically ill patients [25].

It is important to also acknowledge some potential limitations. Firstly, our patient cohort was heterogenous despite strict eligibility criteria. Although we excluded all patients with known pro- or anti-thrombotic conditions, we acknowledge that thrombin generation at baseline pre-CRRT ranged from 7.8 nM to 1255.17 nM, all patients had factor VIII levels above the reference range, and protein C concentrations were below the reference range in the majority of patients. This is likely a reflection of critical illness and multi-organ failure, illustrating the under-recognised prevalence of hypercoagulability in critically ill patients with AKI. However, we only included patients with AKI who needed CRRT and cannot comment on coagulation abnormalities in critically ill patients without AKI. Second, TGA still lacks defined reference values [18]. Consequently, reference ranges for TGA parameters specific to the reagents/analyser/analytical protocol were locally derived from healthy volunteer donors to which patient results were compared. Third, the TGA failed to return a result in 6.8% of samples (8/117). Similarly, the PFA failed to return a result in 26.5% of samples (62/234) due to the test exceeding the maximum permitted run time (300 s). This may be related to the fact that several patients developed thrombocytopenia during the study (4/11) which returns elevated PFA-100 CTs purely as a function of reduced platelet numbers and not necessarily altered function [26]. Although thrombocytopenia was an exclusion criterion, it can obviously develop during the course of an illness. In our study, there was a significantly lower platelet count in those tests that timed out compared to those that returned a result (88 vs. 216, *p* < 0.0001). Fourth, all samples for haemostasis testing were necessarily taken in vacutainers containing 3.2% citrate which leads to citrate concentrations 10.9–12.9 mmol/L and a fall in [Ca^2+^] in the vacutainer to ~ 0. Where analytically necessary, haemostasis assay design principles incorporated replenishment of [Ca^2+^] with 25 mmol/L CaCl_2_ in coagulation screening tests and 15 mmol/L CaCl_2_ in TGA. Consequently, INR, APTT and TGA results were possibly confounded by variations in the final [Ca^2+^]. This was not an issue for specific-component assays that employ calcium replenishment as they were performed to merely assess levels of each parameter and not effects of citrate anticoagulation on their function. Calcium chloride is not replenished in PFA-100 analysis. The citrate anticoagulated samples would therefore not assess any direct effects of citrate in the circulation, but reflect changes in primary haemostasis resulting from the CRRT treatment itself. Fifth, we acknowledge that D-dimer and PFA-100 tests do not reflect the full spectrum of fibrinolysis or platelet function. Global fibrinolysis assays, such as euglobulin clot lysis and dilute clot lysis were not available. Performing specific-component assays like tissue plasminogen activator (t-PA), urokinase-type plasminogen activator (u-PA), plasminogen activator inhibitor-1 (PAI-1), plasminogen, thrombin-activatable fibrinolysis inhibitor (TAFI) or histidine-rich glycoprotein (HRG) was beyond the resources available. However, markers such as t-PA/PAI-1 complexes and plasmin/antiplasmin complexes give broadly the same information as D-dimer. Also, we used widely available assays that could have been applied in this context if our results had suggested that monitoring citrate anticoagulation with them could be valuable. Lastly, the study took 2.5 years to complete and not all planned investigations were performed in all patients [17]. This reflects our very tight inclusion criteria and the challenges of studying coagulation biochemistry in critically ill patients.

Our results have implications for clinical practice and future research. More in-depth investigations of patients with repeated episodes of premature filter clotting are warranted to identify potential contributing factors and to rule out an underlying acquired coagulopathy. This is particularly relevant since filter life has been proposed as a potential marker for quality assessment and performance measurement during CRRT [25]. Finally, the fact that all patients exhibited raised thrombin generation potential should prompt research studies exploring the role of thrombin inhibitors in CRRT.

## Conclusions

Critically ill patients with AKI requiring RRT are hypercoagulable. Citrate does not impact thrombin generation, fibrinolysis or platelet function.

## Methods

### Setting

The study was performed in a 30-bedded multi-disciplinary Critical Care Unit at a tertiary care centre in London, UK, between April 2014 and December 2016.

### Patient population

Critically ill patients in whom the clinical team had decided to start CRRT with RCA were recruited. The inclusion criteria were AKI as defined by the KDIGO criteria [27], age ≥ 18 years, and expectation that CRRT was needed for at least 72 h. To limit confounding by potentially pro- or anti-thrombotic factors, we excluded patients with any of the following: (1) known pre-existing thrombotic or bleeding tendency, including patients with previous thromboembolic or vascular occlusive disease, Factor V Leiden, heparin-induced thrombocytopenia, thrombocytosis, haematological malignancy, systemic lupus erythematosus, antiphospholipid syndrome, haemophilia, thrombocytopenia and liver disease; (2) laboratory evidence of disseminated intravascular coagulation (DIC); (3) transfusion of blood products during the 24 h prior to enrolment; (4) active bleeding; (5) haemoglobin < 75 g/L, haematocrit > 0.55 or platelet count < 100 × 10^9^/L; (6) treatment with an anticoagulant or antiplatelet agent within 7 days prior to enrolment (with exception of unfractionated or low molecular weight heparin for prophylaxis against deep vein thrombosis), or (7) treatment with intravenous heparin within 4 h of study enrolment. To exclude confounding by pre-existing medical conditions that may impact haemostasis, we also excluded patients who were malnourished [body mass index (BMI) < 18.5 kg/m^2^ or unplanned weight loss > 10% of actual body weight (ABW) in the preceding 6 months or BMI < 20 kg/m^2^ with unplanned weight loss > 5% of ABW]. We also excluded patients who received CRRT for an indication other than AKI. We acknowledge that our study involved blood sampling which may contribute to anaemia and therefore excluded patients who may refuse a blood transfusion. Finally, if CRRT was discontinued within 48 h or transfusion of blood products was necessary, participants were withdrawn and a new patient was recruited in their place. This was done to ensure an adequate number of measurements per patient.

### Protocol

Patients received CVVHD using a central dual-lumen dialysis catheter. RCA was delivered as per departmental protocol. Accordingly, 4% tri-sodium citrate (136 mmol/L) was administered into the circuit pre-filter, aiming for an initial citrate concentration of 4 mmol/L in blood and a post-filter ionised [Ca^2+^] in the target range of 0.25–0.34 mmol/L. Subsequent titration of the citrate administration was permitted to maintain the post-filter ionised [Ca^2+^] in target range. Calcium chloride (100 mmol/L) was administered into the return lumen of the dialysis catheter at 0-10 mmol/h to keep the systemic ionised [Ca^2+^] between 1.12 and 1.2 mmol/L.

Prior to commencing CRRT, blood was sampled from the patient's arterial line for measurement of full blood count (FBC) and clotting profile, including prothrombin time expressed as international normalised ratio (INR), activated partial thromboplastin time (APTT) expressed as activated partial thromboplastin time ratio (APTTr), Clauss fibrinogen, D-dimer and platelet function assay (PFA-100). Baseline assessment for hereditary and acquired coagulopathies was performed as previously described [17]. Plasma samples were stored at − 80 °C for subsequent batch testing of thrombin generation.

Following commencement of CRRT, blood samples were taken at 12, 24, 36, 48 and 72 h from an arterial line as well as directly from the CRRT circuit (post-filter) for FBC, INR, APTTr, Clauss fibrinogen, D-dimer, PFA-100, and thrombin generation assay (TGA). FBC samples were transferred to the laboratory in ethylenediaminetetraacetic acid (EDTA) vacutainers whilst all other blood samples were transferred in 3.2% citrate vacutainers.

### Laboratory analyses

The TGA was performed to measure the overall capacity of a plasma sample to form thrombin after initiation of coagulation using TECHNOTHROMBIN® TGA (Technoclone, Vienna, Austria) on a Wallac Victor3 Multilabel Plate Reader (Perkin Elmer, Turku, Finland) to generate total thrombin and time to peak parameters. To generate local reference ranges, the TGA was performed on 24 control samples from healthy volunteers. In these control samples, mean peak thrombin was 195.1 nM (SD 132.0, range 78.1–549.9 nM) and mean time to peak thrombin concentration was 26.6 min (SD 7.3, range 16.2–45.0 min). A PFA-100 analyser (Sysmex UK, Milton Keynes, UK) was used to investigate platelet function using both collagen/ADP and collagen/epinephrine cartridges.

### Data collection

Baseline demographics, including age, gender, height, weight, BMI, comorbidities, reason for ICU admission, and AKI cause were collected.

### Sample size

For this mechanistic study, we aimed for a sample size of at least 10 patients.

### Trial registration

The study was registered on 1st July 2015 with ClinicalTrials.gov (Identifier: NCT02486614). https://clinicaltrials.gov/ct2/show/NCT02486614

### Statistical analysis

Baseline characteristics and coagulation parameters were summarised as frequency (percentage) or mean (SD). Baseline TGA parameters were compared to controls using two sample t-tests. Coagulation and fibrinolysis parameters were summarised as mean (SD) at each time point and mean (95% confidence intervals) and plotted over time along with trajectories of individual patients. Repeated measures analysis of variance (ANOVA) was used to explore changes over time. ANOVAs were limited to measurements up to 48 h as some patients completed CRRT before the 72-h point. Separate ANOVAs were used to compare each of the measurements of blood samples taken from the arterial line from baseline to 48 h and from 12 to 48 h using blood samples from the circuit. Where significant main effects of time were observed, post hoc pairwise companions were carried out using Tukey's honestly significant difference.

Differences in coagulation and fibrinolysis parameters of blood samples taken from the arterial line and the corresponding value obtained from blood drawn from the circuit were plotted against the average of the two parameters. The mean (SD) of the differences was calculated along with the Bland–Altman limits of agreement assuming. Samples drawn from the same patient at different time points were treated as independent. The analysis was carried out using Stata 16 /IC.

## Supplementary Information

**Additional file 1: Figure S1**. Changes in systemic measurements of coagulation over duration of treatment. **Figure S2**. Changes in circuit measurements of coagulation over duration of treatment. **Figure S3**. Bland Altman plots showing difference between systemic and circuit measurements

## Data Availability

The datasets used and analysed during the current study are available from the corresponding author on reasonable request.

## Abbreviations

aβ2GPI: Anti-β2 glycoprotein I antibodies
aCL: Anticardiolipin antibodies
AKI: Acute kidney injury
ANOVA: Analysis of variance
APCR: Activated protein C resistance
APACHE: Acute Physiologic and Chronic Health Evaluation
APC-PCI: Activated protein C-protein C inhibitor
APTTr: Activated Partial Thromboplastin Time ratio
BMI: Body mass index
CRRT: Continuous renal replacement therapy
CT: Closure time
CVVHD: Continuous veno-venous haemodialysis
dAPTT: Dilute activated partial thromboplastin time
dRVVT: Dilute Russell’s viper venom time
INR: International Normalised Ratio
PAI-1: Plasminogen activator inhibitor type 1
PFSCA: Platelet Function Assay using Collagen/Adenosine diphosphate
PFSCE: Platelet Function Assay using Collagen/Epinephrine
RCA: Regional citrate anticoagulation
RRT: Renal replacement therapy
SD: Standard deviation
SOFA: Sequential Organ Failure Assessment
TAT: Thrombin:antithrombin complexes
TGA: Thrombin generation assay
VWF: Von Willebrand factor
VWFAg: Von Willebrand factor antigen
