# Supplementary material for: Effects of regional citrate anticoagulation on thrombin generation, fibrinolysis and platelet function in critically ill patients receiving continuous renal replacement therapy for acute kidney injury: a prospective study
Source: Ann Intensive Care. 2022 Mar 31;12:29. doi: 10.1186/s13613-022-01004-w (PMC8967919; doi:10.1186/s13613-022-01004-w)
Supplement: Supplementary file 1 — Additional file 1: Figure S1. Changes in systemic measurements of coagulation over duration of treatment. Figure S2. Changes in circuit measurements of coagulation over duration of treatment. Figure S3. Bland Altman plots showing difference between systemic and circuit measurements [file 13613_2022_1004_MOESM1_ESM.docx]

**Supplementary Figure 1 Changes in systemic measurements of coagulation over duration of treatment**

**Abbreviations:** INR, International Normalised Ratio; APTTr, Activated Partial Thromboplastin Time ratio; TGA, Thrombin Generation Assay;

PFSCA, Platelet Function Assay using Collagen/Adenosine diphosphate; PFSCE, Platelet Function Assay using Collagen/Epinephrine; CT,

Closure Time.

Repeated measures ANOVAs compared systemic parameters from baseline to 48 hours. Post-hoc pairwise comparisons found significant

differences between haemoglobin at baseline and 36hours (p=0.030) and baseline and 48 hours (p=0.018); platelets at baseline and 36 hours

(p=0.035) and baseline and 48 hours (p=0.040) and INR at baseline and 36hours (p=0.034) and baseline and 48 hours (p=0.024). There were no

significant differences in systemic parameters on CRRT.

**Supplementary Figure 2 Changes in circuit measurements of coagulation over duration of treatment**

**Abbreviations:** INR, International Normalised Ratio; APTTr, Activated Partial Thromboplastin Time ratio; TGA, Thrombin Generation Assay; PFSCA, Platelet Function Assay using Collagen/Adenosine diphosphate; PFSCE, Platelet Function Assay using Collagen/Epinephrine; CT, Closure Time.

Repeated measures ANOVAs of measurements from the circuit compared data from 12hrs to 48hours. Only INR differed overtime, with a significant difference between 12 and 48hrs in posthoc comparisons (p=0.002).

**Supplementary Figure 3 Bland Altman plots showing difference between systemic and circuit measurements**

**Abbreviations:** INR, International Normalised Ratio; APTTr, Activated Partial Thromboplastin Time ratio; TGA, Thrombin Generation Assay; PFSCA, Platelet Function Assay using Collagen/Adenosine diphosphate; PFSCE, Platelet Function Assay using Collagen/Epinephrine; CT, Closure Time.

Lines shown represent the mean differences in systemic and circuit measurements and 95% limits of agreement.
